# Brokering a new path: navigating administrative burdens in the health insurance Marketplaces

**DOI:** 10.1093/haschl/qxag017

**Published:** 2026-01-23

**Authors:** Jessica M Mulligan, David M Anderson, Coleman Drake

**Affiliations:** Department of Anthropology, University of Virginia, Charlottesville, VA 22903, United States; Health Services Policy and Management, University of South Carolina, Arnold School of Public Health, Columbia, SC 29208, United States; Department of Health Policy and Management, University of Pittsburgh, School of Public Health, Pittsburgh, PA 15261, United States

**Keywords:** Affordable Care Act (ACA), health insurance marketplaces, administrative burdens, insurance brokers, uninsurance

## Abstract

Millions will become uninsured when Affordable Care Act enhanced subsidies expire and new, stricter enrollment regulations take effect in 2026. Reductions in federal enrollment assistance that have helped coverage seekers navigate administrative burdens mean that health insurance brokers will be critical in mitigating Marketplace coverage losses. Brokers can be a strong force for maintaining Marketplace enrollment, particularly when the markets they operate in are structured to minimize bad behavior and to maximize outreach. This commentary argues that a robust broker infrastructure is necessary to help Marketplace enrollees retain coverage. We outline how brokers can help people navigate new administrative burdens to Marketplace enrollment and review 2 innovative models for maximizing outreach among people more likely to be uninsured.

## Introduction

Millions of Marketplace enrollees will become uninsured as enhanced premium subsidies expire in 2026. New administrative burdens, including a shortened open enrollment period and income verification requirements,^1^ will further decrease coverage in 2027.^2^ These changes are occurring amidst the Centers for Medicare and Medicaid Services (CMS) drastically reducing federal enrollment assistance that has historically helped coverage seekers navigate administrative burdens.^3^

Strategies for overcoming these new administrative burdens, combined with an effective regulatory structure to prevent fraud, are critical for mitigating insurance loss. This commentary outlines how brokers can help people navigate the new administrative burdens to Marketplace enrollment (see Table 1) and reviews 2 innovative models for maximizing outreach among people more likely to be uninsured.

**Table 1.** Brokers’ potential roles in mitigating new Marketplace administrative burdens.

Administrative burden	Description	Brokers’ role
Learning costs		
Public funding cuts for assisters and advertising outreach (2026)	Assister and navigator programs cut by 90%; federal spending reductions in advertising	Work with health plans, nonprofits, and states to reach populations with high uninsured rates
Understanding increased eligibility requirements (2027)	Enrollees must learn to comply with new eligibility rules, validation processes and income checks (see details under compliance costs below)	Longer interactions with more consumer education
Compliance costs (2027)		
Shortened open enrollment period	Open enrollment period reduced to 6 weeks from November 1 to December 15	Help enrollees enroll in the shorter allowed timespan
Additional income verification	Enrollees must submit income documentation if no tax data are available, in fewer days than previously allowed, or not receive premium subsidies^a^	Help enrollees identify and submit required documentation in timely manner
Active plan selection	Beginning in 2028, enrollees must select a plan each year and will not be able to automatically re-enroll	Leverage established relationships to reach out to enrollees and assist with active re-enrollment
Elimination of low-income special enrollment period	Effective August 2025, enrollees <150% FPL no longer can enroll in coverage outside of enrollment periods	Inform affected enrollees that it is necessary to enroll during open enrollment or have a qualifying life event
Psychological burdens (2026)		
Frustration and anxiety	Enrollees may be surprised by sharp increases in premiums and administrative burdens	Help enrollees identify plans that match their health needs and financial constraints Explain available options to enrollees, guide them through administrative burdens, and provide information about other community sources of care
Fear regarding immigration status	Lawfully present immigrants may avoid enrollment due to fears of legal reprisal	
Financial burdens (2026)		
Enhanced premium tax credit subsidy, eligibility expiration	Enrollees ≤400% FPL will experience premium increases due to reduced subsidies; enrollees >400% FPL will lose subsidy eligibility; some lawfully present immigrants will lose eligibility	Help enrollees identify plans that match their health needs and financial constraints

Funding for assisters and advertising was cut in 2025 by the Centers for Medicare and Medicaid Services, affecting 2026 enrollment. Enhanced premium subsidies from the Inflation Reduction Act expired at the start of 2026. Compliance costs will come into effect in 2027 under the One Big Beautiful Bill Act except for the active plan selection requirement, which will be implemented in 2028, and the end of the low-income special enrollment period, which is already in effect per Centers for Medicare and Medicaid Services administrative changes.

^a^Enrollees automatically re-enrolling from zero-premium plans must pay a small positive premium until their subsidy eligibility has been verified.

Abbreviation: FPL, federal poverty level.

## Broker-assisted enrollment is already the most common form of assisted enrollment

Health insurance brokers are critical in mitigating Marketplace coverage losses. Brokers are well positioned to navigate administrative burdens to enrollment, as they already facilitated over 70% of active enrollment in the 30 HealthCare.gov states in 2024.^4^ Brokers, state-licensed insurance professionals, are paid by insurers through commissions for enrolling consumers in Affordable Care Act (ACA) coverage. These payments, usually $15–$30 per member per month, are factored into premiums. Brokers’ role in the Marketplaces significantly expanded after CMS implemented Enhanced Direct Enrollment (EDE) for HealthCare.gov in 2018. Enhanced Direct Enrollment substantially reduced the time for brokers to sign enrollees up for coverage. Brokers’ Marketplace involvement further increased in 2021 as enrollment surged with the creation of enhanced premium subsidies.^4^ That brokers already have relationships with most Marketplace enrollees puts them in a uniquely advantageous position to help navigate administrative burdens.

## Concerns with broker-assisted enrollment

Broker payment models that incentivize volume and new customer bonuses can be vulnerable to fraud and abuse. This issue arose as brokers’ role in the Marketplaces expanded, prompting 274 000 complaints to CMS in 2023.^5^ One scheme in Florida defrauded the federal government of nearly $140 million by enrolling lower-income people in coverage without their knowledge or consent.^6^ Fraudulent broker schemes primarily occurred in HealthCare.gov states, which have minimal broker regulations, making it easier for bad actors to misuse EDE.

The CMS implemented new regulations in 2024 that were highly successful in reducing fraudulent enrollment.^7,8^ These changes included requiring 3-way phone calls between the enrollee, the broker, and a HealthCare.gov representative before processing significantly altered applications. Initial efforts to combat fraud reduced reports of fraudulent enrollment by 30% and lowered the rate of plan switching initiated by brokers and agents by 70%.^7^ Despite these successes and the fact that state-based Marketplaces with robust broker regulations largely did not experience fraudulent enrollment, broker fraud has been used to justify strict rule changes and proposals to eliminate zero premium plans.^9^

There also are concerns that brokers are less likely to assist clients who need language assistance, earn lower incomes, or do not have reliable internet access.^10^ Brokers, paid by enrollee-month, may be incentivized to avoid potential enrollees with complex and time-consuming cases.

Creating broker programs with guardrails to prevent fraud and engage people having difficulty with administrative burdens is critical for maintaining Marketplace enrollment. We document 2 initiatives that could serve as models for achieving these goals.

## Connecticut's broker academy

Connecticut's state-based Marketplace, Access Health CT,^11^ has created an innovative broker academy linked to expanding coverage to populations with high uninsurance rates. Access Health CT recruits brokers from communities with the highest coverage barriers and supports new brokers through training by sponsoring testing to obtain licensing, providing laptops, and pairing new brokers with experienced mentors. The collaborative relationship with the Marketplace persists through referrals including warm handoffs, ongoing training and feedback, and joint participation in enrollment events. Enrollment specialists at Access Health CT rely on brokers’ specialized plan knowledge and awareness of local provider networks to support enrollees in choosing a plan. Eligibility questions are typically handled directly by the Marketplace. Brokers are empowered to leverage knowledge of insurance and their communities to best match enrollees to coverage. Access Health CT actively monitors potentially fraudulent enrollment and claims, although much potential fraud is eliminated before enrollment occurs because of the state's broker licensing requirements.

Connecticut's small size and historic role in the insurance industry contribute to this model's success, but other state marketplaces can also capitalize on their existing broker workforce to create similar initiatives that partner with and train brokers from communities with the highest coverage barriers.

## The Palmetto Project

The Palmetto Project is a multifaceted nonprofit community organization focused on education and health care in South Carolina. The organization originally operated with a navigator contractor, InsureSC, but when their enrollment assistance program was defunded in 2017 they became a nonprofit insurance agency.

Palmetto offers a “no wrong door approach” for Medicaid, Medicare, and Marketplace coverage. Its staff, known as “resource specialists,” are salaried, licensed insurance brokers, which mitigates the financial incentive to prefer plans that pay a higher commission.^12^ The Palmetto Project's services are provided at no cost to the clients. Funding is provided by enrollment commissions.

Nonprofit brokerages such as the Palmetto Project engage in community outreach by combining institutional knowledge, community trust, and a sustainable funding model from commissions. Palmetto Project's ability to help people find the right type of coverage is particularly valuable for lower-income enrollees who often face the greatest administrative burdens, including Medicaid coverage churn and limited health insurance literacy.

Start-up costs are a barrier to establishing nonprofit brokerages. Exams, licensing fees, and background checks can total $1000 per employee, depending on the state. Nonetheless, entry costs are relatively low and commission revenue streams may currently be more dependable than grant funding. With a small investment of seed money, community organizations that formerly housed navigators or assisters could retool under this model.

## Conclusion

Brokers are well positioned to help mitigate ongoing Marketplace coverage losses due to increased administrative burdens. While bad actors among brokers have facilitated fraudulent enrollment in years past, the federal government took effective steps to reduce fraud in 2024. Brokers have helped achieve historic low uninsurance rates. Brokers can continue to be a strong, positive force for maintaining Marketplace enrollment, particularly when the markets they operate in are structured to minimize bad behavior and to maximize outreach.

State governments should emulate Connecticut by recruiting brokers from communities with the highest coverage barriers; training brokers and supporting them through the process so it is not burdensome; and taking an active anti–fraud-monitoring role. Nonprofit and community organizations, meanwhile, can partner with brokers in the model of the Palmetto Project. Hiring brokers on a salary basis reduces incentives to steer enrollees towards plans that do not serve their needs, and having a “no wrong door” approach helps to reduce uninsurance broadly.

As more states continue to establish their own state-based Marketplaces, it will be essential that they structure them to leverage brokers’ expertise to support legitimate enrollment in the Marketplace. When new administrative burdens are implemented in 2027, a robust broker infrastructure will be necessary to help at-risk Marketplace enrollees retain coverage.

## Supplementary Material

qxag017_Supplementary_Data
