# Supplementary material for: Brokering a new path: navigating administrative burdens in the health insurance Marketplaces
Source: Health Aff Sch. 2026 Jan 23;4(2):qxag017. doi: 10.1093/haschl/qxag017 (PMC12898919; doi:10.1093/haschl/qxag017)
Supplement: qxag017_Supplementary_Data [file qxag017_supplementary_data.docx]

| \| ICMJE DISCLOSURE FORM \| \| \| --- \| --- \| \| **Date:** \| 11/17/2025 \| \| **Your Name:** \| Jessica Mulligan \| \| **Manuscript Title:** \| Brokering a New Path: Navigating Administrative Burdens in the Health Insurance Mar-ketplaces \| \| **Manuscript Number (if known):** \| Click or tap here to enter text. \| \| In the interest of transparency, we ask you to disclose all relationships/activities/interests listed below that are related to the content of your manuscript. “Related” means any relation with for-profit or not-for-profit third parties whose interests may be affected by the content of the manuscript. Disclosure represents a commitment to transparency and does not necessarily indicate a bias. If you are in doubt about whether to list a relationship/activity/interest, it is preferable that you do so.  The author’s relationships/activities/interests should be defined broadly. For example, if your manuscript pertains to the epidemiology of hypertension, you should declare all relationships with manufacturers of antihypertensive medication, even if that medication is not mentioned in the manuscript.  In item #1 below, report all support for the work reported in this manuscript without time limit. For all other items, the time frame for disclosure is the past 36 months. \| \|  \|  \| \| \| **Name all entities with whom you have this relationship or indicate none (add rows as needed)** \| **Specifications/Comments (e.g., if payments were made to you or to your institution)** \| \| --- \| --- \| --- \| --- \| --- \| \| **Time frame: Since the initial planning of the work** \| \| \| \| \| \| **1** \| All support for the present manuscript (e.g., funding, provision of study materials, medical writing, article processing charges, etc.)  **No time limit for this item.** \| \| \|  \| **None** \| \| --- \| --- \|  \| Agency for Healthcare Research and Quality (AHRQ), U.S. Department of Health and Human Services, under grant number R01HS030083 \| Grant funding that supports the research \| \| --- \| --- \| \| University of Virginia start up funds \| Start up funding as part of a new faculty package that supports the article processing charges \| \|  \| Click the tab key to add additional rows. \| \| \| \| **Time frame: past 36 months** \| \| \| \| \| \| **2** \| \| Grants or contracts from any entity (if not indicated in item #1 above). \| \|  \| **None** \| \| --- \| --- \|  \| Supporting the Health Care Workforce during Emergencies and Disasters: Improving Dissemination through Rapid Ethnographic Research Methods. Marion and Jasper Whiting Foundation. 2023-2024. $7750 \| Funding to travel to the UK to study with the Rapid Evaluation Lab at University College London \| \| --- \| --- \| \| Collaborative Research: RUI: Ethics of Care and Compounded Disaster: Transformation in Puerto Rico after Maria. Co-PI, Adriana M Garriga-López Kalamazoo College. National Science Foundation. NSF Award Number: 2049568. $192,938 (Providence College). 03/01/2021 – 2/29/2024 \| Research project not related to the article under submission. \| \|  \|  \| \| \| \| **3** \| \| Royalties or licenses \| \|  \| **None** \| \| --- \| --- \|  \|  \|  \| \| --- \| --- \| \|  \|  \| \|  \|  \| \| \| \| **4** \| \| Consulting fees \| \|  \| **None** \| \| --- \| --- \|  \|  \|  \| \| --- \| --- \| \|  \|  \| \|  \|  \| \|  \|  \| \| \| \| **5** \| \| Payment or honoraria for lectures, presentations, speakers bureaus, manuscript writing or educational events \| \|  \| **None** \| \| --- \| --- \|  \| AHRQ panelist for their 35^th^ anniversary celebration, October 2024 \| Received a $300 honoraria for participating \| \| --- \| --- \| \|  \|  \| \|  \|  \| \| \| \| **6** \| \| Payment for expert testimony \| \|  \| **None** \| \| --- \| --- \|  \|  \|  \| \| --- \| --- \| \|  \|  \| \|  \|  \| \| \| \| **7** \| \| Support for attending meetings and/or travel \| \|  \| **None** \| \| --- \| --- \|  \| Support from Providence College where I was a faculty member until July 2025 to attend academic conferences. \|  \| \| --- \| --- \| \|  \|  \| \|  \|  \| \| \| \| **8** \| \| Patents planned, issued or pending \| \|  \| **None** \| \| --- \| --- \|  \|  \|  \| \| --- \| --- \| \|  \|  \| \|  \|  \| \| \| \| **9** \| \| Participation on a Data Safety Monitoring Board or Advisory Board \| \|  \| **None** \| \| --- \| --- \|  \|  \|  \| \| --- \| --- \| \|  \|  \| \|  \|  \| \| \| \| **10** \| \| Leadership or fiduciary role in other board, society, committee or advocacy group, paid or unpaid \| \|  \| **None** \| \| --- \| --- \|  \|  \|  \| \| --- \| --- \| \|  \|  \| \|  \|  \| \| \| \| **11** \| \| Stock or stock options \| \|  \| **None** \| \| --- \| --- \|  \|  \|  \| \| --- \| --- \| \|  \|  \| \|  \|  \| \| \| \| **12** \| \| Receipt of equipment, materials, drugs, medical writing, gifts or other services \| \|  \| **None** \| \| --- \| --- \|  \|  \|  \| \| --- \| --- \| \|  \|  \| \|  \|  \| \| \| \| **13** \| \| Other financial or non-financial interests \| \|  \| **None** \| \| --- \| --- \|  \|  \|  \| \| --- \| --- \| \|  \|  \| \|  \|  \| \| \| \|  \| \|  \|  \| \| \| **Please place an “X” next to the following statement to indicate your agreement:** \| \| \| \| \| \|  \| \| I certify that I have answered every question and have not altered the wording of any of the questions on this form. \| \| \|  ICMJE DISCLOSURE FORM | |
| --- | --- | --- | --- | --- | --- | --- | --- | --- | --- | --- | --- | --- | --- | --- | --- | --- | --- | --- | --- | --- | --- | --- | --- | --- | --- | --- | --- | --- | --- | --- | --- | --- | --- | --- | --- | --- | --- | --- | --- | --- | --- | --- | --- | --- | --- | --- | --- | --- | --- | --- | --- | --- | --- | --- | --- | --- | --- | --- | --- | --- | --- | --- | --- | --- | --- | --- | --- | --- | --- | --- | --- | --- | --- | --- | --- | --- | --- | --- | --- | --- | --- | --- | --- | --- | --- | --- | --- | --- | --- | --- | --- | --- | --- | --- | --- | --- | --- | --- | --- | --- | --- | --- | --- | --- | --- | --- | --- | --- | --- | --- | --- | --- | --- | --- | --- | --- | --- | --- | --- | --- | --- | --- | --- | --- | --- | --- | --- | --- | --- | --- | --- | --- | --- | --- | --- | --- | --- | --- | --- | --- | --- | --- | --- | --- | --- | --- | --- | --- | --- | --- | --- | --- | --- | --- | --- | --- | --- | --- | --- | --- | --- | --- | --- | --- | --- | --- | --- | --- | --- | --- | --- | --- | --- | --- | --- | --- | --- | --- | --- | --- | --- | --- | --- | --- | --- | --- | --- | --- | --- | --- | --- | --- | --- | --- | --- | --- | --- | --- | --- | --- | --- | --- | --- | --- | --- | --- | --- | --- | --- | --- | --- | --- | --- | --- |
| **Date:** | 11/18/25 |
| **Your Name:** | David Anderson |
| **Manuscript Title:** | Click or tap here to enter text. |
| **Manuscript Number (if known):** | Click or tap here to enter text. |
| In the interest of transparency, we ask you to disclose all relationships/activities/interests listed below that are related to the content of your manuscript. “Related” means any relation with for-profit or not-for-profit third parties whose interests may be affected by the content of the manuscript. Disclosure represents a commitment to transparency and does not necessarily indicate a bias. If you are in doubt about whether to list a relationship/activity/interest, it is preferable that you do so.  The author’s relationships/activities/interests should be defined broadly. For example, if your manuscript pertains to the epidemiology of hypertension, you should declare all relationships with manufacturers of antihypertensive medication, even if that medication is not mentioned in the manuscript.  In item #1 below, report all support for the work reported in this manuscript without time limit. For all other items, the time frame for disclosure is the past 36 months. | |

|  | | | **Name all entities with whom you have this relationship or indicate none (add rows as needed)** | **Specifications/Comments (e.g., if payments were made to you or to your institution)** |
| --- | --- | --- | --- | --- |
| **Time frame: Since the initial planning of the work** | | | | |
| **1** | All support for the present manuscript (e.g., funding, provision of study materials, medical writing, article processing charges, etc.)  **No time limit for this item.** | | \|  \| **None** \| \| --- \| --- \|  \| Commonwealth Fund \| Research grant (Co-I) \| \| --- \| --- \| \| AHRQ R01 \| Research Grant consultant \| \|  \| Click the tab key to add additional rows. \| | |
| **Time frame: past 36 months** | | | | |
| **2** | | Grants or contracts from any entity (if not indicated in item #1 above). | \|  \| **None** \| \| --- \| --- \|  \| Pharmaceutical Care Management Association – Foundation \| Research grant Medicare Part D (MPI) \| \| --- \| --- \| \| Commonwealth Fund \| Research grant (Co-I) \| \| AHRQ R01 \| Research Grant consultant \| | |
| **3** | | Royalties or licenses | \|  \| **None** \| \| --- \| --- \|  \|  \|  \| \| --- \| --- \| \|  \|  \| \|  \|  \| | |
| **4** | | Consulting fees | \|  \| **None** \| \| --- \| --- \|  \| Alliant Health Plan \| Personal Consulting fees – ACA strategy \| \| --- \| --- \| \| EVENSUN LLC \| Personal Consulting fees – ACA strategy \| \| MEAPTA LLC \| Personal Consulting fees – unrelated ACA work \| \| Pharmaceutical Care Management Association \| Research consulting fees Medicare Part D \| | |
| **5** | | Payment or honoraria for lectures, presentations, speakers bureaus, manuscript writing or educational events | \|  \| **None** \| \| --- \| --- \|  \|  \|  \| \| --- \| --- \| \|  \|  \| \|  \|  \| | |
| **6** | | Payment for expert testimony | \|  \| **None** \| \| --- \| --- \|  \|  \|  \| \| --- \| --- \| \|  \|  \| \|  \|  \| | |
| **7** | | Support for attending meetings and/or travel | \|  \| **None** \| \| --- \| --- \|  \| AHIP \| Baltimore MD 2025 \| \| --- \| --- \| \| Texas Association of Health Plans \| Austin 2024 \| \| Health Plan Alliance \| Dallas 2022 & 2023 \| \| Arnold Ventures \| Washinton National Harbor 2024 \| | |
| **8** | | Patents planned, issued or pending | \|  \| **None** \| \| --- \| --- \|  \|  \|  \| \| --- \| --- \| \|  \|  \| \|  \|  \| | |
| **9** | | Participation on a Data Safety Monitoring Board or Advisory Board | \|  \| **None** \| \| --- \| --- \|  \|  \|  \| \| --- \| --- \| \|  \|  \| \|  \|  \| | |
| **10** | | Leadership or fiduciary role in other board, society, committee or advocacy group, paid or unpaid | \|  \| **None** \| \| --- \| --- \|  \|  \|  \| \| --- \| --- \| \|  \|  \| \|  \|  \| | |
| **11** | | Stock or stock options | \|  \| **None** \| \| --- \| --- \|  \|  \|  \| \| --- \| --- \| \|  \|  \| \|  \|  \| | |
| **12** | | Receipt of equipment, materials, drugs, medical writing, gifts or other services | \|  \| **None** \| \| --- \| --- \|  \|  \|  \| \| --- \| --- \| \|  \|  \| \|  \|  \| | |
| **13** | | Other financial or non-financial interests | \|  \| **None** \| \| --- \| --- \|  \|  \|  \| \| --- \| --- \| \|  \|  \| \|  \|  \| | |
|  | |  |  | |
| **Please place an “X” next to the following statement to indicate your agreement:** | | | | |
|  | | I certify that I have answered every question and have not altered the wording of any of the questions on this form. | | |

| ICMJE DISCLOSURE FORM | |
| --- | --- |
| **Date:** | 11/17/25 |
| **Your Name:** | Coleman Drake |
| **Manuscript Title:** | Brokering a New Path: Navigating Administrative Burdens in the Health Insurance Mar-ketplaces |
| **Manuscript Number (if known):** | Click or tap here to enter text. |
| In the interest of transparency, we ask you to disclose all relationships/activities/interests listed below that are related to the content of your manuscript. “Related” means any relation with for-profit or not-for-profit third parties whose interests may be affected by the content of the manuscript. Disclosure represents a commitment to transparency and does not necessarily indicate a bias. If you are in doubt about whether to list a relationship/activity/interest, it is preferable that you do so.  The author’s relationships/activities/interests should be defined broadly. For example, if your manuscript pertains to the epidemiology of hypertension, you should declare all relationships with manufacturers of antihypertensive medication, even if that medication is not mentioned in the manuscript.  In item #1 below, report all support for the work reported in this manuscript without time limit. For all other items, the time frame for disclosure is the past 36 months. | |

|  | | | **Name all entities with whom you have this relationship or indicate none (add rows as needed)** | **Specifications/Comments (e.g., if payments were made to you or to your institution)** |
| --- | --- | --- | --- | --- |
| **Time frame: Since the initial planning of the work** | | | | |
| **1** | All support for the present manuscript (e.g., funding, provision of study materials, medical writing, article processing charges, etc.)  **No time limit for this item.** | | \|  \| **None** \| \| --- \| --- \|  \| Agency for Health Research and Quality (AHRQ) \| R01HS030083 \| \| --- \| --- \| \|  \|  \| \|  \| Click the tab key to add additional rows. \| | |
| **Time frame: past 36 months** | | | | |
| **2** | | Grants or contracts from any entity (if not indicated in item #1 above). | \|  \| **None** \| \| --- \| --- \|  \| Commonwealth Foundation \|  \| \| --- \| --- \| \| National Institute on Drug Abuse \|  \| \| Hillman Foundation \|  \| | |
| **3** | | Royalties or licenses | \|  \| **None** \| \| --- \| --- \|  \|  \|  \| \| --- \| --- \| \|  \|  \| \|  \|  \| | |
| **4** | | Consulting fees | \|  \| **None** \| \| --- \| --- \|  \|  \|  \| \| --- \| --- \| \|  \|  \| \|  \|  \| \|  \|  \| | |
| **5** | | Payment or honoraria for lectures, presentations, speakers bureaus, manuscript writing or educational events | \|  \| **None** \| \| --- \| --- \|  \| Keio University (Japan) \|  \| \| --- \| --- \| \| Cato Institute \|  \| \| Ergo Global Intelligence \|  \| \| Stephenson Institute for Classical Liberalism \|  \| \| Weill Cornell Medical College \|  \| | |
| **6** | | Payment for expert testimony | \|  \| **None** \| \| --- \| --- \|  \|  \|  \| \| --- \| --- \| \|  \|  \| \|  \|  \| | |
| **7** | | Support for attending meetings and/or travel | \|  \| **None** \| \| --- \| --- \|  \| Joachim-Herz Foundation \|  \| \| --- \| --- \| \|  \|  \| \|  \|  \| | |
| **8** | | Patents planned, issued or pending | \|  \| **None** \| \| --- \| --- \|  \|  \|  \| \| --- \| --- \| \|  \|  \| \|  \|  \| | |
| **9** | | Participation on a Data Safety Monitoring Board or Advisory Board | \|  \| **None** \| \| --- \| --- \|  \|  \|  \| \| --- \| --- \| \|  \|  \| \|  \|  \| | |
| **10** | | Leadership or fiduciary role in other board, society, committee or advocacy group, paid or unpaid | \|  \| **None** \| \| --- \| --- \|  \|  \|  \| \| --- \| --- \| \|  \|  \| \|  \|  \| | |
| **11** | | Stock or stock options | \|  \| **None** \| \| --- \| --- \|  \|  \|  \| \| --- \| --- \| \|  \|  \| \|  \|  \| | |
| **12** | | Receipt of equipment, materials, drugs, medical writing, gifts or other services | \|  \| **None** \| \| --- \| --- \|  \|  \|  \| \| --- \| --- \| \|  \|  \| \|  \|  \| | |
| **13** | | Other financial or non-financial interests | \|  \| **None** \| \| --- \| --- \|  \|  \|  \| \| --- \| --- \| \|  \|  \| \|  \|  \| | |
|  | |  |  | |
| **Please place an “X” next to the following statement to indicate your agreement:** | | | | |
|  | | I certify that I have answered every question and have not altered the wording of any of the questions on this form. | | |
